# Hypervirulent *Streptococcus agalactiae* septicemia in twin ex-premature infants transmitted by breast milk: report of source detection and isolate characterization using commonly available molecular diagnostic methods

**DOI:** 10.1186/s12941-020-00396-6

**Published:** 2020-11-26

**Authors:** Edward P. C. Ager, Eric D. Steele, Lindsey E. Nielsen, Matthew A. Nestander, Katrin Mende, Steven E. Spencer

**Affiliations:** 1 Department of Clinical Investigations, Brooke Army Medical Center, San Antonio, TX USA; 2 Department of Pathology and Area Laboratory Services, Brooke Army Medical Center, San Antonio, TX USA; 3 Department of Pediatric Infectious Disease, Brooke Army Medical Center, San Antonio, TX USA; 4 Infectious Disease Clinical Research Program, Preventive Medicine and Biostatistics Department, Uniformed Services University of the Health Sciences, Bethesda, MD USA; 5 Henry M. Jackson Foundation for the Advancement of Military Medicine, Inc, Bethesda, MD USA; 6 Infectious Disease Service, Brooke Army Medical Center, San Antonio, TX USA

**Keywords:** GBS, Neonatal, Mastitis

## Abstract

**Background:**

Group B *Streptococcus* (GBS) infections caused by *Streptococcus agalactiae* is a leading cause of meningitis and sepsis in neonates, with early-onset GBS symptoms emerging during the first week of life and late-onset occurring thereafter. Perinatal transmission of GBS to the neonate through the birth canal is the main factor associated with early-onset neonate infections, while less is understood about the source of late-onset infections.

**Methods:**

In this report we describe a case of twin ex-premature infants who presented one month after birth with GBS septicemia. The mother had been appropriately screened at gestational age 35–37 weeks and laboratory methods failed to detect GBS colonization by culture or clinical molecular methods. In attempts to identify and isolate the source of GBS infection, additional surveillance swabs were collected from the mother at the time of neonate admission. Culture and a commercially available, FDA-cleared molecular PCR assay were performed.

**Results:**

No GBS was detected from swabs collected from the perianal, thigh/groin or axillary areas. However, expressed breast milk and swabs from the breastmilk pump were positive by both methods. Since simultaneous culture and molecular methods which used breastmilk as a source were performed, investigators ascertained the limit of detection for GBS in breastmilk. The limit of detection was determined to be tenfold lower than that of LIM-broth enriched cultures—the FDA-approved source. Subsequent whole genome sequencing (WGS) analysis of isolates recovered from breastmilk and blood cultures from the infants demonstrated all strains were related and characterized as ST-452. Both infants responded very well to treatment and continued to have no related events or concerns at the two-year follow up appointment.

**Conclusions:**

Strain type 452 (capsular type IV) has recently emerged as a hypervirulent strain and has previously been documented as causing GBS infections in elderly populations. Antibiotic therapy resolved both mother and infant infections. Subsequent testing for the presence of GBS in breastmilk samples also showed an absence of bacteria. This is the first report of infant twins late-onset GBS infections caused by the hypervirulent *S. agalactiae* ST-452 with breastmilk as the source.

## Background

*Streptococcus agalactiae* (GBS) infections have been associated with severe invasive infections in newborns since the first cases of puerperal fever were described in the 1930s [1]. GBS is characterized as a colonizing microbe which impacts 30% of pregnant women. There are two distinguishable clinical syndromic phases: early-onset disease occurring within the first week of life (usually within the first 24 h), and late-onset disease presenting after 7 days of age (7 to 90 days postpartum). Early onset disease is caused by vertical transmission of GBS from a colonized mother to her newborn: through either ascending infection from the genital tract or GBS transmission to the newborn during labor and birth. It is estimated 50% of colonized mothers may pass GBS to their fetus. Reports show early onset disease occurs in 1% of colonized newborns usually after onset of labor or rupture of membranes. Published Case series report fatality rates as high as 50%, with pneumonia and meningitis described as the leading clinical syndromes. In contrast, late onset disease occurs after exposure of GBS to the infant from the mother or other sources after the first week of life and up to 3 months [2]. Breast milk, nosocomial or community acquired infections have been the sources most often associated with late onset disease in case reports. Prematurity is a common risk factor [2].

Currently, intervention to prevent late onset disease is through intravenous administration of antibiotics (IAP). Reports estimate the pooled incidence of neonatal morbidity and mortality to be approximately 0.49 per 1000 including pre-term, still birth and neonatal GBS associated incidence worldwide [3].

In 1992, guidelines were implemented to examine all women at 35–37 weeks gestation and identify carriers so that appropriate prophylaxis during childbirth can be administered. Prenatal screening by molecular and culture based methods, plus antibiotic therapy, have reduced the prevalence of early onset GBS infections from 1.6/1000 to 0.22/1000 live births from 1989 to 2016 [4]. However, prenatal screening had no significant effect on the prevalence rate of 0.24/1000 births for late-onset (7–89 days) GBS infections [5].

The source of these late-onset infections is debated but often presumed—like many infectious diseases—to be transmitted from the mother due to the neonate-maternal relationship [6]. Maternal milk has previously been suggested as a possible source of infection and is often underestimated as a specimen source due to the lack of consideration during clinical case investigations.

Despite the ease of breastmilk collection and specimen submission to the clinical laboratory, current FDA-approved assays are not approved for the use of breastmilk as a testing matrix. This limits the ability to screen suspected sources. Nonetheless, many reports have identified contaminated breast milk as a likely cause of single and/or recurrent episodes of GBS infections [7–10]. GBS, like many gram-positive cocci*,* are common skin microbes and are known to colonize many body sites [2]. This favors the possibility that infant suckling or transfer from breastmilk could be a possible route of infection. Interestingly, no correlation is found between GBS colonized peripartum women and subsequent presence of GBS in breastmilk. In one study, all positive cases were from GBS-negative women who were screened by swabs and plate cultures during their pregnancies [11]. Furthermore, GBS-breastmilk positivity rates are low with estimates between 0.8–3.5%, depending on the detection method used [11, 12]. Yet, another case report suggested that the incidence of GBS in breastmilk may be even higher and is dependent on the sensitivity of the testing method [13].

Mastitis is believed to increase the possibility of GBS infections even if the mastitis remains subclinical [14]. Reports so far suggest mothers with mastitis have higher bacterial GBS counts than those without mastitis [15]. Recommendations differ on whether breast-feeding should be interrupted during mastitis, breastmilk pasteurized or antibiotic treatment for GBS colonization be initiated on the mother and/or her infant [16]. Support of the transfer of GBS from the mother-infant relationship has been demonstrated. One particular study showed 18 infants who were either a twin or triplet (31% of the surveilled group) were co-infected—with the exception of 2 siblings [17]. Infant GBS colonization may be more common than realized and infection onset is influenced by innate immunity and physiological barriers; such as, integrity of mucosa layers, immune status in infants, overall bacterial load and GBS strain virulence. All *S. agaclatiae* isolates carry common virulent factors, such as pili and capsular polysaccharides (CPS) [18, 19]. Six et al. described another hypervirulent clone, ST-17, associated with invasive meningitis [19]. Another recently discovered hypervirulent clone, clonal complex 17 (CC17), also produced the HvgA surface adhesion protein along with a type III CPS [20]. The HvgA protein is required for translocation through the intestinal barrier with tissue tropism for the meninges [21]. Changes in CPS led to the characterization of CPS serotype IV hypervirulent strain type 452 [22, 23].

GBS strain ST-452 has been documented in elderly GBS infections but has not yet been documented in neonates or infants. Laboratory detection of GBS by traditional culture has less sensitivity than PCR detection methods yet, this methodology remains the standard methodology used for GBS detection in many clinical microbiology laboratories possibly due to cost limitations [24]. Breastmilk is not a commonly accepted source for GBS molecular detection, partially because of the lack of information on the composition of breastmilk and how compositional differences can interfere with detection. Additionally, molecular limits of detection (LOD) equivalency studies are not readily available.

In this study, we present a case of dichorionic diamniotic infants that were delivered via C-section at 30 + 1 weeks due to preterm labor and developed late onset GBS infection and developed neonatal sepsis. Maternal serologies were notable for a negative GBS swab. Initial hospitalization was relatively unremarkable until day of life 33 when twin A began having multiple desaturation events. A complete sepsis evaluation revealed GBS bacteremia. Four days later, twin B also became symptomatic with similar events. Her blood culture also grew GBS. The infants were still in the NICU when the infection occurred. They were being fed primarily fresh expressed maternal breast milk (one child was able to take some small feeds by mouth rather than strictly through NG feeds). The maternal milk was being fortified with formula but was not pasteurized due to the significant loss of nutritional value caused by this process. Maternal expressed breast milk was cultured and grew GBS as well. A short time thereafter, the mother was diagnosed with mastitis. Investigation revealed improper sterilization of breastmilk pump and supplies, with a culture resulting in GBS growth. All GBS isolates were identified as the same organism via pulsed-field gel electrophoresis. The infection was caught by the hospital staff very quickly after onset and therefore did not result in significant clinical deterioration in either child.

In this study we showed the identification and characterization of a unique GBS clone from specimens obtained from: the Mother, twins, collected breast milk and the breast pump device used by patients who at the time of the study was negative for mastitis. We also compared LOD of the Cepheid GBS Direct PCR assay using breastmilk as a matrix versus the FDA-approved standard LIM- broth media procedure.

## Materials and methods

Standard pediatric blood cultures were drawn following the clinical suspicion of symptoms consistent with sepsis. Infant B’s blood was drawn for culture on day of life (DOL) 33 and Infant A’s blood was drawn DOL 37 for incubation in the Bac-T Alert culture system from bioMérieux at 35–37 °C. After incubation, the resulting pathogen identification was confirmed by bioMérieux VITEK MALDI-TOF and BioFire PCR methods. Antepartum and peripartum swab surveillance samples were enriched in LIM-broth and analyzed using the Cepheid Xpert GBS LB PCR assay. This clinically approved assay was used to detect the 3′ DNA region adjacent to the *cfb* gene of *S. agalactiae*. Cepheid PCR of breastmilk was completed using both direct and enriched (indirect) sample sources (Additional file 1).

Estimates of GBS concentration and the assay’s limit of detection was determined for breastmilk by introducing known concentrations of GBS bacteria into PCR-negative breastmilk. To determine if the GBS was localized in the milk ducts or if the mother was otherwise colonized, the pediatric Infectious Disease team obtained specimens from groin, axilla, breast, and perirectal sites. The perirectal swab was processed for GBS cultures according to current clinical screening protocols for ascertaining GBS colonization, a process which includes LIM broth enrichment. The other samples were brought to the lab separately and since only one swab was obtained for each sample those swabs were used for LIM broth enrichment only. The enriched broth was then processed according to the manufacturer’s guidelines.

A Medela breast pump used by the mother was brought to the lab. Samples were collected as follows: First, the pump assembly had 20 mL of sterile water poured through to rinse the entire surface. Second, the resulting rinsate was collected in 50 mL conical tubes. Each breast cup was similarly sampled. The four collection bottles had 20 mL of sterile water poured inside. The bottle was gently swirled so that the entire surface area was rinsed. Approximately 1 mL of rinsate was transferred into LIM broth and cultured overnight at 35–37 °C. The enriched broth was then processed according to the manufacturer’s guidelines.

In an effort to evaluate breast milk that the mother had previous pumped and frozen, samples were transported to the lab to be evaluated for possible GBS contamination. The milk was thawed at room temperature and then evaluated using two methods. First, two swabs were swirled in each milk sample. One swab was put in LIM broth for enrichment and the other was evaluated using a direct GBS method. The enriched broth was then processed according to the manufacturer’s guidelines. The positive samples were then plated to 5% sheep blood trypticase soy agar (blood agar) plates using a 1 µL aliquot and struck for quantification.

Breast Milk Quantitation: The two positive breast milk samples were thawed at room temperature, and then a 1 µL inoculation loop was used to inoculate a blood agar plate, streaking for quantity. There were 8 beta (β) hemolytic colonies on the plate from the sample labeled DOL 33 and 10 colonies on the sample labeled DOL 37. Each colony was then sub-cultured on to a blood agar plate for identification. The colonies were then evaluated by using VITEK MALDI-TOF.

All isolates underwent DNA extraction using QIAamp DNA Mini Kit (QIAGEN, Hilden, Germany) and subsequently clonality was assessed by pulsed-field gel electrophoresis (PFGE) analysis. PFGE was performed based on the previous publication by McEllistrem and colleagues with a few slight modifications [25]. Specifically, the pulse times used in this study were 3.5 to 25 s for 12 h and 1 to 5 s for 8 h. In addition, whole genome sequencing was performed by the Multidrug-Resistant Organism Repository and Surveillance Network (MRSN). Sequences were submitted to GENBANK Accession no. 13397758, ASM808603v1.

## Results

**Fig. 1:**
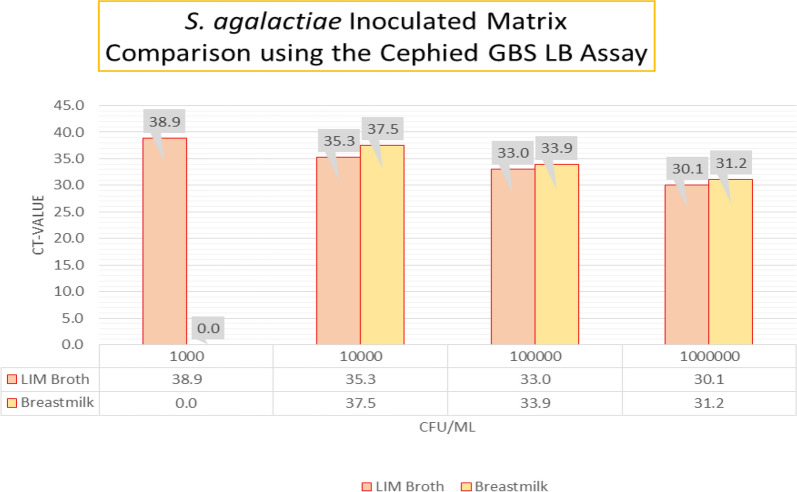
*S. agalaciae* inoculated Matrix Compared using the Cepheid GBS PCR Assay The results verify that Breastmilk and LIM Broth show similar LOD for GBS

Antenatal PCR screening using LIM-broth enriched cultures failed to detect GBS upon labor presentation. Twins were born at 30 weeks gestation via C-section and received a rule-out sepsis evaluation and 48 h of antibiotic prophylaxis. At age 35 days, the male twin became symptomatic with subsequent pediatric blood cultures growing GBS. The female twin became symptomatic five days later prompting additional PCR surveillance of the mother’s groin, axilla, breast, and perirectal areas, but all specimens collected and tested were negative. The mother’s breastmilk, however, was GBS positive despite no clinical evidence of mastitis as of day 35 postpartum. Indirect and direct PCR of stored breastmilk collected from 18–40 days postpartum detected GBS at day 35 and 40 with no samples provided in between. Quantitation of organisms directly from breastmilk at day 35 and 40 was estimated to be 8,000 and 10,000 CFU/mL, respectively. The mother developed clinical mastitis on day 36. GBS was also detected in the breast milk pump attachments and collection bottles. Using breastmilk as a direct matrix, the limit of detection of the assay was tenfold higher than (103 CFU/mL) that of LIM-enriched cultures (Fig. 1). Internal controls remained valid at all known CFU concentrations suggesting breastmilk does not inhibit PCR assay utility.

All β-hemolytic colonies were determined to be *S. agalactiae* with 99.9% confidence conferring an approximate CFU/mL of 8,000 for the DOL 33 milk sample and 10,000 for the DOL 37 milk samples.

**Fig. 2:**
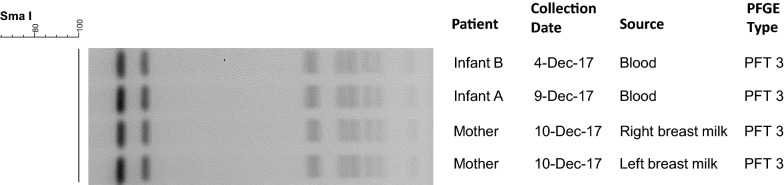
Pulsed-field gel electrophoresis (PFGE) of *Streptococcus agalactiae* isolated from blood cultures and breastmilk samples collected from the mother and twins. The PFGE analysis showed that all four isolates are identical, they are the same strain

Pulsed field gel electrophoresis (PFGE) identified the four isolates of *S. agalactiae* that was isolated from the blood. The PFGE demonstrated that all four isolates were identical (Fig. 2).

**Fig. 3:**
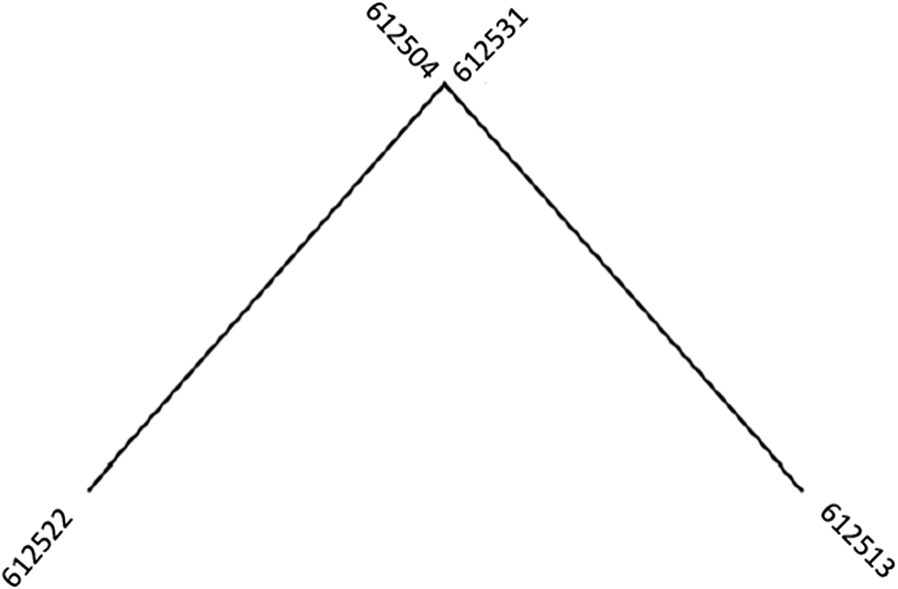
Dendrogram depicting phylogenic relationship between the four *Streptococcus agalactiae* isolates

Whole genome sequencing, performed by MRSN, determined that all four *S. agalactiae* isolates were nearly identical with the greatest separation of sequences being only two single nucleotide polymorphisms (SNPs). In silico multi-locus sequence typing (MLST) and serotyping assigned all the isolates to ST-452 and serotype IV, respectively (Table 1). A difference of zero-two SNPs across the entire genome is consistent with person-to-person spread (Table 2). A dendrogram depicting the relationship between all four isolates is shown in Fig. 3.

**Table 1:** Basic characteristics of the isolates

MRSN ID	Patient	Source	MLST	Serotype
612504	Male twin (A)	Blood	452	IV
612513	Female twin (B)	Blood	452	IV
612522	Mother	Fluid	452	IV
612531	Mother	Fluid	452	IV

**Table 2:** Phylogenetic relationship between the isolates

SNP matrix	612504	612513	612522	612531
612504	0			
612513	1	0		
612522	1	2	0	
612531	0	1	1	0

The number of SNPs separating each isolate is displayed in the matrix

## Discussion

The association of breast milk with postpartum onset of infant GBS disease has been rarely reported [11]. With the recent technological advances in detection of GBS by diverse molecular methods, more cases have been reported [11, 26].

In agreement with previous reports [17, 27–29], this case report indicates that breast-milk is a potential source of GBS neonatal LOD infection. Infected milk could well be a determinant for late- onset GBS disease which may be underestimated and potentially ignored. Bacteria surveillance on breast milk should be performed whenever a breast-fed infant, especially a premature neonate, presents with suspicion of late-onset sepsis given that breastfed preterm infants, are more susceptible to invasive infections. Despite the ability to detect surface or perirectal GBS colonization using enriched PCR, these twins were positively diagnosed with their mother’s breastmilk as a source. The use of clinical PCR methods for detection of GBS directly from breastmilk is not routinely practiced and subsequently the sensitivity and specificity of these methods have not been established.

In our study, direct and indirect methods were concordant suggesting that breastmilk specimens can be a reliable and appropriate medium for direct GBS detection with a similar assay limit of detection as LIM broth specimens. Furthermore, this case suggests direct perinatal screening of breastmilk for GBS, in the absence of routine surveillance methods and prior to the clinical onset of mastitis, has clinical utility. Previously, culture based studies for GBS in breastmilk did not yield positive results.

Even though the patients in this study required a small and brief increase in respiratory support during the acute illness stage as well as prolonged IV access for antibiotic administration (specifically 10 days). Ultimately, both infants responded well to treatment and were discharged home with their parents. No further concerns have arisen since discharge through their two-year follow-up appointment.

## Conclusion

There is evidence in the literature to support the routine if not standardized submission of breast milk for clinical use and epidemiological studies. The molecular studies contained in this case report demonstrate clonality between the isolates collected from all 3 patients across multiple source types and indicate a clear pattern of increased LOD as disease progression occurs. Based on our study we propose that breast milk can and should be used as a reliable specimen source for standardized molecular assays to characterize late onset GBS disease.

## Supplementary information

**Additional file 1. ***S. agalactiae* Inoculated Matrix Comparison using the Cephied GBS LBAssay.

## Data Availability

All relevant data are within the paper.

## Abbreviations

GBS: Group B *Streptococcus*
LOD: Limit of detection
MLST: Multi-locus sequence typing
MRSN: Multidrug resistant organism repository
PFGE: Pulsed field gel electrophoresis
