# Supplementary material for: Hypervirulent Streptococcus agalactiae septicemia in twin ex-premature infants transmitted by breast milk: report of source detection and isolate characterization using commonly available molecular diagnostic methods
Source: Ann Clin Microbiol Antimicrob. 2020 Nov 26;19:55. doi: 10.1186/s12941-020-00396-6 (PMC7691097; doi:10.1186/s12941-020-00396-6)
Supplement: Supplementary file 1 — Additional file 1. S. agalactiae Inoculated Matrix Comparison using the Cephied GBS LBAssay. [file 12941_2020_396_MOESM1_ESM.pdf]

ADDITIONAL FILE:

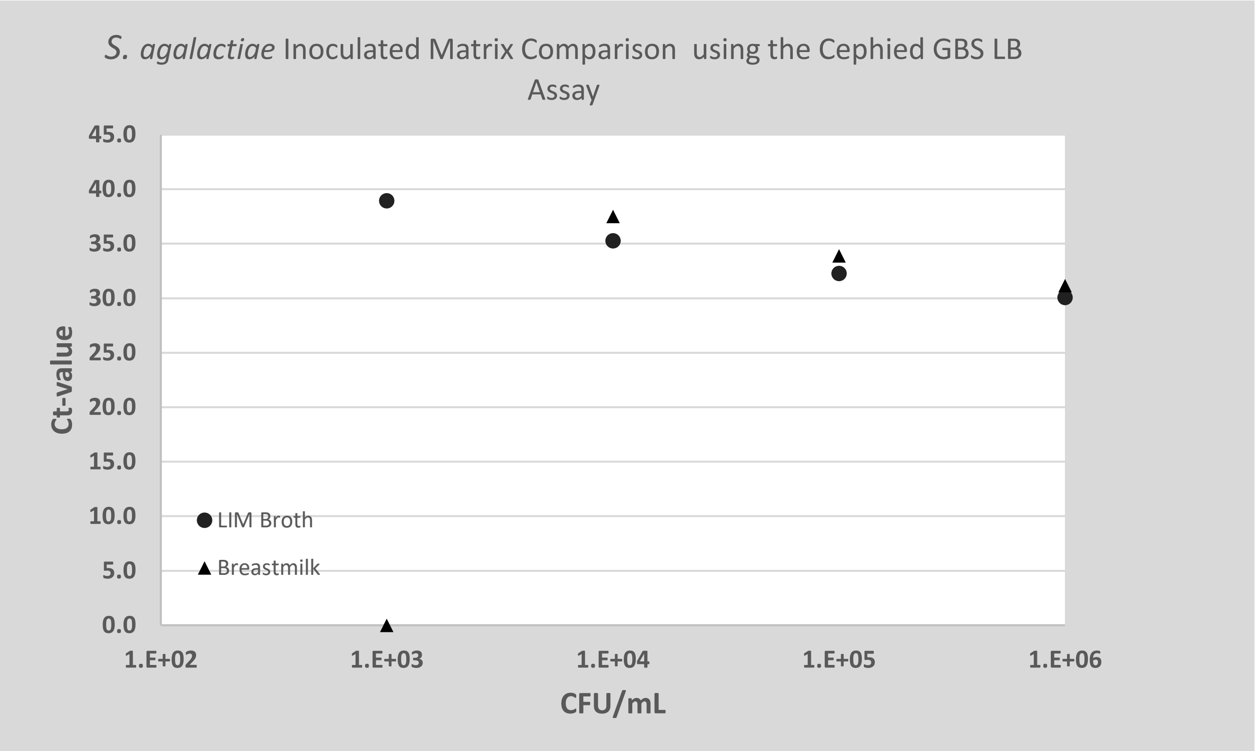

Comparison of Ct-values using serially diluted GBS in LIM broth and Breastmilk run on the Cepheid Genexpert.

	LIM Broth				
CFU/mL	Replicate 1	Replicate 2	Replicate 3	average	STDEV
1000	40.4	37.2	39.2	38.9	1.6
10000	35.2	34.2	36.4	35.3	1.1
100000	33.5	33.1	32.4	33.0	0.6
1000000	29.1	29.6	31.5	30.1	1.3
	Breastmilk				
CFU/mL	Replicate 1	Replicate 2	Replicate 3	average	STDEV
1000	0	0	0	0.0	0.0
10000	36.4	36.8	39.3	37.5	1.6
100000	34	33.4	34.3	33.9	0.5
1000000	30.8	31	31.7	31.2	0.5
